# Progression of Clinical Features in Lewy Body Dementia Can Be Detected Over 6 Months

**DOI:** 10.1212/WNL.0000000000012450

**Published:** 2021-09-07

**Authors:** Elie Matar, Simon R. White, John-Paul Taylor, Alan Thomas, Ian G. McKeith, Joseph P.M. Kane, Ajenthan Surendranathan, Glenda M. Halliday, Simon J.G. Lewis, John T. O'Brien

**Affiliations:** From the Department of Psychiatry (E.M., S.R.W., A.S., J.T.O.) and MRC Biostatistics Unit (S.R.W.), University of Cambridge, UK; Forefront Parkinson's Disease Research Clinic (E.M., G.M.H., S.J.G.L.) and Brain and Mind Centre (E.M., G.M.H., S.J.G.L.), Faculty of Medicine and Health, University of Sydney, Australia; Newcastle Translational and Clinical Research Institute (J.-P.T., A.T., I.G.M.), Campus for Ageing and Vitality, Newcastle University, Newcastle Upon Tyne; and Centre for Public Health (J.P.M.K.), Queen's University Belfast, UK.

## Abstract

**Objective:**

This study aimed to quantify the trajectory and magnitude of change of the key clinical features and corresponding symptom domains of dementia with Lewy bodies (DLB) and Parkinson disease dementia (PDD), including global cognition, parkinsonism, recurrent visual hallucinations, cognitive fluctuations, and sleep disturbance.

**Methods:**

One hundred sixteen patients with Lewy body dementia (DLB = 72, PDD = 44) underwent assessment at baseline and 3 and 6 months as part of a prospective multicenter randomized controlled trial. Linear mixed models were constructed for core outcome measures using the Mini-Mental State Examination (MMSE), motor section of the Unified Parkinson's Disease Rating Scale (UPDRS-III), Dementia Cognitive Fluctuations Scale (DCFS), and Neuropsychiatric Inventory (NPI).

**Results:**

Within the time frame of our study (6 months), we were able to identify a significant cognitive decline of 1.3 points on the MMSE (*p* = 0.002) and significant worsening of motor parkinsonism with an increase in UPDRS-III score of 3.2 points (*p* = 0.018). Fluctuation severity also increased using the DCFS with a 6-month change in score of 1.3 points (*p* = 0.001). Uniquely, a signal for increased severity of sleep symptoms of 1.2 points (NPI-sleep) was also detectable (*p* = 0.04). Significant changes in neuropsychiatric symptoms were not detected. There was no difference in rates of change of scores between DLB and PDD.

**Discussion:**

Clinically significant rates of change in core clinical features can be detected and quantified in Lewy body dementia over a relatively short period (6 months) using common clinical instruments and thus may be useful as clinical endpoints for therapeutic trials of disease-modifying and symptomatic agents.

Dementia with Lewy bodies (DLB) and Parkinson disease dementia (PDD), referred to collectively as Lewy body dementia, are characterized by the presence of dementia accompanied by shared clinical features, including motor parkinsonism, recurrent visual hallucinations, cognitive fluctuations, and REM sleep behavior disorder.^1-3^

Despite the significant public health impact,^4-7^ major challenges in the field have resulted in a relative paucity of symptomatic and disease-modifying trials in DLB and PDD.^8^ One such challenge is the delineation of appropriate outcome measures for tracking disease progression and severity. Indeed, few prospective studies have directly investigated the natural history of key clinical features in Lewy body dementia.^6,9-13^ Existing studies have focused mainly on changes in cognitive outcomes^9-11,14-16^ with little known about the natural history of neuropsychiatric features,^17^ motor parkinsonism, fluctuations, and sleep symptoms, especially over the 6-month time frames typical of clinical trials.^18,19^ Such information would be critical for the incorporation of these features as endpoints in future clinical trials.

To address this gap, we sought to quantify the trajectory and magnitude of change of the key clinical features and symptom domains of cognitive impairment, motor parkinsonism, cognitive fluctuations, and neuropsychiatric and sleep disturbances in a cohort of patients with PDD and DLB over a period of 6 months using widely used clinical rating scales. We analyzed data from a prospective multicenter nonpharmacologic intervention trial.^20^ The nature of the intervention, designed to encourage expected standards of care, made it ideal for assessing the natural progression of clinical features under the conditions of a clinical trial.

## Methods

### Participants

**Figure 1:**
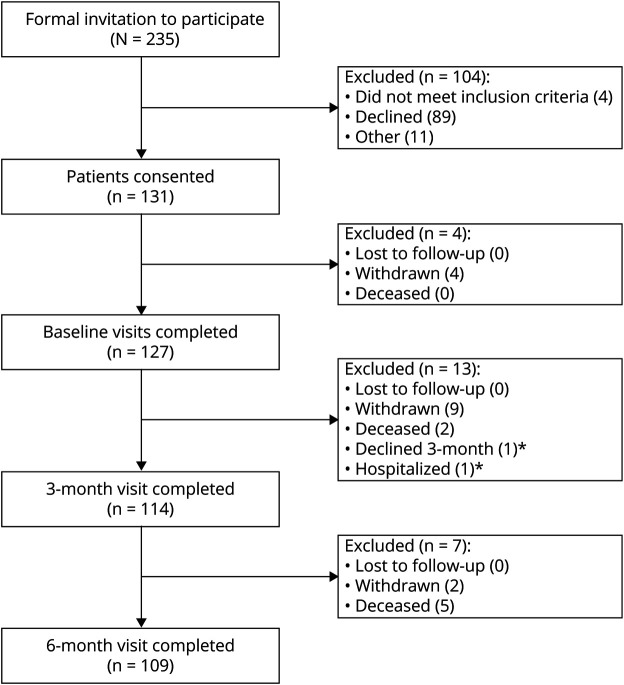
Consolidated Standards of Reporting Trials Diagram Including Enrollment, Registration, and Rates of Completion of Study *Two participants who were not able to make the 3-month visit were subsequently able to make the 6-month visit.

Data from 127 participants with Lewy body dementia (77 DLB, 50 PDD) were analyzed from a cluster randomized trial (Diagnosis and Management of Neurodegenerative Dementia [DIAMOND-Lewy] Study; ISRCTN11083027)^20^ performed between 2016 and 2017 in 23 memory or movement disorder services across 8 main jurisdictions (trusts) of the UK National Health Service (NHS; 4 trusts in North East England and 4 trusts in East Anglia). All patients underwent clinical assessment and were diagnosed as having probable DLB or PDD according to current consensus criteria.^2,3^ Half of the services were randomized to receive a management toolkit comprising a summary of current evidence-based guidelines^21^ for symptomatic treatment of Lewy body dementia (made freely available since the conclusion of the study), while the other services continued with standard care (control arm). From these, 131 participants were recruited to be assessed at baseline and 3 and 6 months. Of those recruited, 127 participants underwent baseline assessments (Figure 1). Because this study assessed the rate of change, data for at least 2 time points were required to contribute to the group-level trajectories. Therefore, patients who could not be followed up after the baseline measurement (n = 11) were excluded, leaving 116 participants (72 DLB, 44 PDD) for the final analysis (including 107 patients with data for all 3 time points, 7 patients with data at baseline and 3 months, and 2 patients with data at baseline and 6 months). Results of the trial with respect to the main intervention have recently been published.^20^

### Standard Protocol Approvals, Registrations, and Patient Consents

Ethics approval for this study was obtained from the NHS Research Ethics Committee. Written informed consent was obtained from all participants and their caregivers/next of kin.

### Clinical Variables

All baseline and 3- and 6-month assessments were conducted by the same research team members in each respective region (North East England and East Anglia, UK) blinded to the service allocation (toolkit or standard care). Core clinical features were assessed with commonly used and validated research instruments. Cognition was measured using the Montreal Cognitive Assessment (MoCA) and the MMSE. Motor parkinsonism was graded by use of Section III of the Movement Disorder Society Unified Parkinson's Disease Rating Scale (UPDRS-III).^22^ Cognitive fluctuations were assessed with the Dementia Cognitive Fluctuation Scale (DCFS).^23^ Neuropsychiatric symptoms were assessed with the Neuropsychiatric Inventory (NPI).^24^ The NPI assesses 12 behavioral domains according to an interview with the caregiver. To maximize the ability to detect a rate of change in this variable, the trajectories of neuropsychiatric symptoms were analyzed for 3 predetermined scores derived from the NPI: the total NPI score summed across all 12 items; the NPI hallucinations score (NPI-hallucinations; frequency × severity); and a 4-item subscore (NPI-4) calculated as the sum of scores for 4 items of hallucinations, delusions, depression, and apathy.^25^ The NPI-4 has been identified in a previous study to consist of items sensitive to Lewy body dementia and has been used as a primary efficacy endpoint in previous randomized trials.^19,26^ Sleep disturbance was also analyzed with the sleep subscore of the NPI (NPI-sleep) as a patient-centered and clinically relevant indicator of sleep quality.

All medications and their dosages were recorded at each visit. Over the course of the trial, 3 patients (2.6%; 3 control, 0 intervention) had changes in the dose/type of antipsychotic medication, 20 patients (17.2%; 8 control, 12 intervention) had changes in dopaminergic medication, and 21 patients (18.1%; 13 control, 8 intervention) had changes in acetylcholinesterase inhibitors.

### Statistical Analysis

All statistical analyses were performed with the IBM Statistical Package for the Social Sciences (version 26.0.0, IBM Corp, Armonk, NY). Descriptive statistics (regarding baseline) are presented as mean (SD) or frequency (percentage). While accounted for in the mixed modeling below, baseline comparison between DLB and PDD with respect to variables of interest was performed with a 2-tailed independent-samples *t* test or χ^2^ test as appropriate (α level = 0.05).

To explore longitudinal changes of the variables of interest, we applied linear mixed-effects modeling using time as a fixed effect and intercept and slope as random effects. This statistical approach was felt to be best suited to account for the natural heterogeneity in the severity of features at baseline and individual variation in longitudinal trajectories.^27^ The outcomes of interest (dependent variables) included the scores derived from the MMSE, MoCA, UPDRS-III, DCFS, NPI (total), NPI-4, NPI-hallucinations, and NPI-sleep. A linear mixed-effect model was constructed for each outcome of interest. An individualized set of prespecified fixed effects (in addition to time) were included in each model constituting confounding variables pertaining to the outcome of interest (specified in the Results section). Although there was no significant difference between intervention and control groups at baseline or follow-up in any of the above outcomes of interest, the binarized intervention status (control vs management toolkit) was included as a fixed effect in all models to account for any potential contribution. Because the primary hypothesis was that we would expect longitudinal progression in the variables of interest, time (in months) was included as a continuous, fixed effect in all models with time 0 occurring from the date of baseline measurement. Random intercepts and slopes were grouped by individual and fitted with a normal distribution around a zero mean. The Wald test was used to test for the significance of random effects in the models. Model fitting was achieved with the maximum likelihood estimation method. Interaction terms between time and intervention and between time and diagnosis were included as fixed effects in initial models for each outcome variable and removed from the final model when they did not achieve significance. On the basis of an a priori statistical plan to account for confounders/covariates, the main-effects (noninteraction) terms were retained in the final models because they were presumed to be still relevant to the parameter estimates of interest (even if not statistically significant). Exploratory models were simplified only when a significant fixed effect was detected (exploratory and final models included in eAppendix 1). All supplementary materials, including eAppendix 1, eAppendix 2, and eFigure 1, are available from Dryad (doi.org/10.5061/dryad.9zw3r22dz).

### Data Availability

Deidentified participant data are available to investigators with appropriate data transfer agreements and institutional board approval.

## Results

### Baseline Characteristics

Group characteristics (DLB vs PDD) are provided in Table 1. There were no significant differences in age, sex, and proportion of patients in the intervention arm between diagnostic groups. However, as expected, patients with PDD had higher severity of motor parkinsonism as scored on UPDRS-III and were taking higher doses of dopaminergic medications.

**Table 1:**
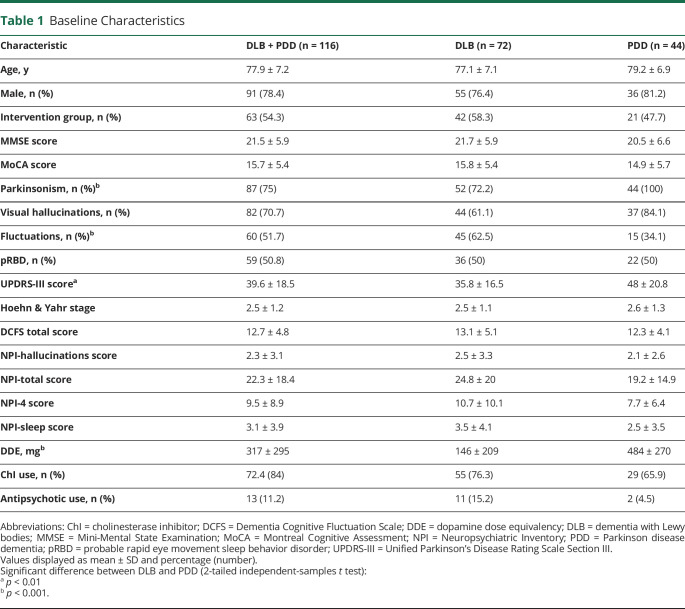
Baseline Characteristics

### Cognitive Decline

**Figure 2:**
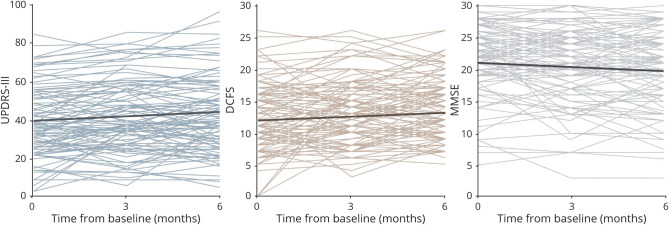
Clinical Variables With Significant Progression Over 6 Months Group-level linear mixed models of fixed effects overlaid onto data for variables in which a significant change in time was detected. Linear trajectory (bold line) is displayed as a function only of time in months from baseline. Mean group variables have been inputted in the model for the other fixed effects within each model (e.g., age, dopaminergic dose). Unified Parkinson's Disease Rating Scale Section III (UPDRS-III) and Dementia Cognitive Fluctuation Scale (DCFS) scores increased significantly over the 6 months of the study, while Mini-Mental State Examination Score (MMSE) score showed a significant decline.

Within the time frame of our study, longitudinal cognitive decline was significantly detected with the MMSE (Figure 2 and Table 2). Covariates included age, cholinesterase use, intervention allocation, and diagnosis (PDD or DLB). The group decline of MMSE score over 6 months was −1.3 points (95% confidence interval [CI] −2.2, −0.5; *p* = 0.002) with an annualized rate of change of −2.7 points (95% CI −4.3, −1.0). MoCA scores also showed a strong trend toward a decline with time, although this was not statistically significant (*p* = 0.06).

**Table 2:**
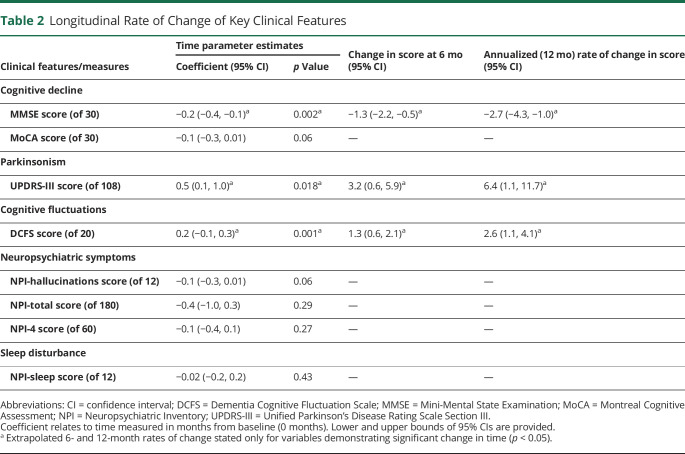
Longitudinal Rate of Change of Key Clinical Features

### Parkinsonism

Using a linear mixed model including diagnosis, intervention, age, and levodopa equivalent dose (UPDRS performed in “on” state), we were able to detect a significant change in time in UPDRS-III score across the sample (Figure 1 and Table 2). The estimated rate of change in UPDRS-III score was 3.2 points over 6 months (95% CI 0.6, 5.9; *p* = 0.018), with a corresponding annualized change of 6.4 points (95% CI 1.1, 11.7). There was no significant interaction between presence or absence of Parkinsonism at baseline and change in Parkinsonism over time (eAppendix 2 [doi.org/10.5061/dryad.9zw3r22dz]).

### Cognitive Fluctuations

A significant increase in DCFS with time was detected over the course of the study (Figure 1 and Table 2) with the use of linear models that covaried for diagnosis, intervention, and cholinesterase use (*p* = 0.001). The 6-month increase on the DCFS scale was estimated to be 1.3 points (95% CI 0.6, 2.1) with an annualized rate of change of 2.6 points (95% CI 1.1, 4.1). We did not find a significant effect of any other variables included in the model. There was no significant interaction between presence or absence of cognitive fluctuations at baseline and change in time of the DCFS score (e-appendix 2 [doi.org/10.5061/dryad.9zw3r22dz]).

### Neuropsychiatric Features

Linear mixed models for 3 measures of neuropsychiatric symptoms (NPI-total, NPI-hallucinations score, and NPI-4) were constructed considering intervention, diagnosis, use of cholinesterase inhibitors, use of antipsychotics, and age as covariates (eAppendix 1 [doi.org/10.5061/dryad.9zw3r22dz]). A small proportion of individuals (n = 10) were prescribed additional antipsychotic medications throughout the course of the study. No significant change in time was detected for any of the 3 neuropsychiatric symptom measures (Table 2). Plots of the individual patient scores revealed significant intraindividual variability (eFigure 1 [doi.org/10.5061/dryad.9zw3r22dz]). There was a trend toward reduced NPI-hallucination severity score (*p* = 0.06) over the time course of this study. However, the small magnitude of change and CIs crossing zero suggests that this was unlikely to be clinically meaningful. There was no significant interaction between the presence or absence of hallucinations at baseline and any of the neuropsychiatric measures (eAppendix 2 [doi.org/10.5061/dryad.9zw3r22dz]).

### Sleep Disturbance

In our initial model, we were unable to detect any significant change in NPI-rated severity of sleep disturbance over the course of this study (Table 2). Covariates included age, diagnosis, intervention, antipsychotic use (sedating actions), and use of cholinesterase inhibitors (adverse reactions with insomnia in some patients). In a post hoc analysis exploring a potential interaction between the presence of sleep disturbance at baseline and change in sleep scores, we found a significant group-level change in sleep disturbance severity over time (0.2 points per month; 95% CI 0.34, 0.76; *p* = 0.04, eAppendix 2 [doi.org/10.5061/dryad.9zw3r22dz]). Furthermore, the presence of sleep disturbance at baseline (NPI-sleep score >0, n = 57) conferred an extra 0.55-point worsening per month (95% CI 0.34, 0.76) compared to those without sleep disturbance at baseline.

### Individual Variability in Trajectory of Measures (Random Effects)

Interparticipant variation in the baseline and longitudinal trajectory of the core features was represented through the inclusion of intercept and time (grouped by individuals) as random effects in the models above. Variance estimates of the random effects for each core feature expressed in SDs are shown in Table 3. Relative to the magnitude of the group-level baseline measures (Table 1) and time fixed-effect estimates (Table 2), large interindividual variation in baseline scores was seen across all measures. Significant interindividual variation in the trajectory of features was also noted for measures of cognitive decline and parkinsonism.

**Table 3:**
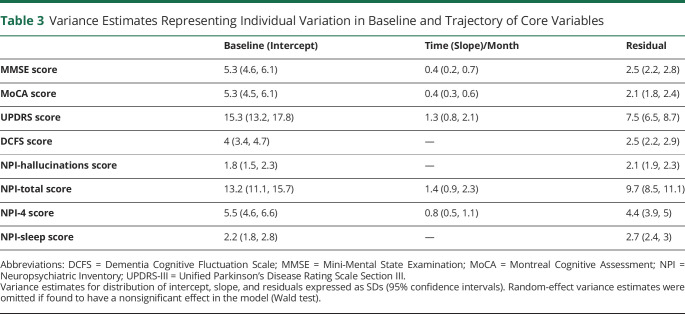
Variance Estimates Representing Individual Variation in Baseline and Trajectory of Core Variables

### Effect of Diagnosis

Overall, no significant difference was detected between patients with DLB and those with PDD on the rate of progression of core clinical features in any of the mixed models. However, it is worth noting that despite the lack of significance, the estimates of the effect of diagnosis as a fixed effect did tend to shift the linear trajectory in an expected direction (e.g., higher UPDRS-III score in PDD; higher DCFS score in DLB; see supplementary materials [doi.org/10.5061/dryad.9zw3r22dz]).

## Discussion

The ability to detect and quantify longitudinal change of key clinical features of Lewy body dementia is essential for understanding the natural history of this disorder and the planning of therapeutic trials. Using common clinical instruments, we have been able to explicitly characterize and quantify the trajectory of several core features in a large cohort of patients with DLB and PDD. Specifically, over the time course of only 6 months, we found it possible to detect significant worsening of motor parkinsonism, cognitive fluctuations, and cognitive decline but not neuropsychiatric symptoms. Furthermore, we were able to quantify the high degree of interindividual variability in clinical trajectories between patients while also showing that, within the time frame of the study, trajectories of these clinical features between PDD and DLB are comparable.

In our study, we were able to detect a significant longitudinal decline using the MMSE. Our annualized rate of decline in MMSE score is consistent with a recent international multicenter cohort study consisting of >1,000 patients reporting a mean annual decline of 2.1 points in DLB and 1.8 points in PDD using 3 annual measurements.^11^ A high degree of interparticipant variability has been noted in rate of MMSE score decline in patients with DLB compared with patients with Alzheimer disease (AD)^9,28^ and is consistent with the random effects reported in Table 3 showing an SD of 0.4 points per month around the group-level estimate of −0.2 points per month. As discussed below, significant variability was also seen with the MoCA. This inherent variability may reflect the vulnerability of neuropsychometric measures to the fluctuating cognition characteristic of Lewy body dementia patients (discussed further below). It also highlights the challenge of translating trial methodologies used in other dementias, which are often biased solely toward cognitive outcomes, to Lewy body dementias.^8^ However, previous longitudinal studies in Lewy body dementia emphasize a faster but more variable annual rate of decline in MMSE scores compared to AD.^9-11,14,15^ In line with this, our data demonstrate that, despite this variability, MMSE may be a feasible measure of cognitive decline even over a relatively short time frame, demonstrating significant group-level changes over a period of 6 months using 3 time points.

Although the MoCA has been shown to be more sensitive in detecting cognitive impairment in patients with Lewy body dementia,^29^ our results suggest that this tool may be less suited as a measure of change for longitudinal tracking of cognition in trials. As seen here, MoCA scores tend to be consistently lower than MMSE scores, which may be associated with floor effects in the evaluation of changes over time. Furthermore, MoCA scores have been shown to be associated with higher individual variability at baseline compared to MMSE scores in patients with Lewy body dementia.^10^ Thus, while MoCA has significant diagnostic value, its prognostic limitations may restrict its utility as a marker of cognitive decline in longitudinal trials.

The UPDRS is the most frequently used outcome measure in symptomatic trials of patients with Parkinson disease.^30^ More recently, with the emergence of disease-modifying trials, there is also growing use of the UPDRS motor score as an endpoint to track disease progression.^31^ We found that, even considering levodopa use and baseline variation in scores, a significant change in motor parkinsonism rated with the UPDRS-III could be estimated, corresponding to an increase of 3.2 points over 6 months and an annualized rate of change of 6.4 points. These values are comparable to the previously determined minimal clinically important change of 5 points on the UPDRS motor in trials of Parkinson disease.^32,33^ Furthermore, the increase in UPDRS-III score at 6 months in Lewy body dementia exceeds the annual rate of change of 2.4 points per year reported in early untreated Parkinson disease.^34^ While the minimal clinically important change has yet to be specifically defined for Lewy body dementia, the comparatively rapid rate of change reported in our study supports parkinsonism as a feasible outcome measure for clinical trials assessing the impact of disease-modifying therapies in Lewy body dementias.

In this study, we were able to systematically investigate the progression of cognitive fluctuations. We found a significant group-level increase of 1.3 points in DCFS score over 6 months, with an annualized rate of change of 2.6 points over 12 months. The DCFS is a clinically validated rating scale demonstrating good sensitivity, specificity, and test-retest and interrater reliabilities.^23^ The present version of the scale is based on the summed responses of items from the original scale found to best discriminate between patients with and without cognitive fluctuations (i.e., marked differences in functioning during the daytime, daytime somnolence, daytime drowsiness, and altered levels of consciousness during the day).^23^ A minimal difference in score constituting a clinically meaningful change to patients and their caregivers has yet to be determined for the DCFS. The modest size differences at the group level may relate to the psychometric properties of the scale itself. It may also be accounted for by the considerable variability in the expression of this symptom between patients, noting some patients did not report symptoms of cognitive fluctuations during the study (Figure 1). Furthermore, the lack of significance of including time as a random effect in our cohort suggests that there was less interindividual variability in the progression of this marker and that fluctuation symptoms may track more uniformly between patients than other clinical measures. Cognitive fluctuations have recently been reported to occur in high frequency in the mild cognitive impairment stage of DLB.^35^ Thus, as we move toward earlier diagnosis and recruitment of patients into disease-modifying trials, our findings highlight the potential utility of fluctuation severity as an outcome measure even in prodromal populations.

We were unable to detect any significant progression in hallucinations and other composite measures of psychiatric symptoms using the NPI over the time course of our study. Neuropsychiatric manifestations across all dementias are highly variable in their prevalence and expression.^36^ In Lewy body dementias, psychiatric symptoms such as visual hallucinations are present in up to 80% of patients and regarded as a core diagnostic feature for the disorder,^2^ and the absence of quantifiable progression needs to be reconciled with the accepted impact of neuropsychiatric symptoms on quality of life, caregiver burden, and risk of institutionalization.^37,38^ It is likely that many neuropsychiatric features reach a certain level of prominence before diagnosis with only modest further deterioration detected in the time course of this study. This is reflected by high baseline measures of NPI symptoms (Table 1) and is consistent with a recent study of patients with mild dementia that found a high proportion of reported neuropsychiatric symptoms at baseline and only modest changes in absolute NPI scores over 5 years.^39^ Alternatively, the lack of a significant change in continuous measures of hallucinations may suggest that categorical variables (e.g., absence or presence) might be a more appropriate means of capturing symptom change over time. In line with this, a recent longitudinal study comparing categorical state changes of neuropsychiatric symptoms between DLB and AD over a 12-year period found that patients with DLB were more likely to exhibit a relapsing/remitting nature rather than continuous worsening or improvement.^17^ Currently, there is no scale designed specifically for measuring visual hallucinations in DLB, and our results highlight the need to develop such a scale or instead consider testing alternative scales validated in other populations such as the Psychosis and Hallucinations Questionnaire^40^ or the North-East Visual Hallucinations Interview.^41^

RBD is a core diagnostic feature of DLB,^2^ and sleep disturbances more generally are increasingly recognized as an important symptom complex of Lewy body disorders.^42,43^ The longitudinal progression of sleep disturbances in Lewy body dementias has not been explicitly investigated. In our study, the NPI-sleep was used as a pragmatic caregiver and patient-centered measure of overall burden of sleep disturbances, including, but not limited to, disruptive dream enactment behaviors. We found that there was a significant change in the burden of sleep disturbance over the 6-month time frame, which was present only once the interaction between the presence of sleep disturbance and time was taken into account. This finding suggests that there is a detectable worsening of sleep disturbances over time in DLB and PDD. Further prospective studies using questionnaires targeting various aspects of sleep (e.g., insomnia, fragmentation, nocturia) and assessment of RBD specifically (such as through the use of sleep diaries^44^) will be required to understand the trajectory of this symptom complex and its feasibility as an endpoint in symptomatic and disease-modifying clinical trials in Lewy body dementia.

DLB and PDD share many clinical, neurochemical, and pathologic features, distinguished by the timing of onset of cognitive symptoms relative to parkinsonism.^45,46^ Such distinction results in the differential expression of core symptoms at baseline (Table 1). However, PDD and DLB were not significant covariates in the group-level trajectory of any core symptom and did not interact with time. This is aligned with previous studies finding no significant difference between the groups in relation to cognitive decline,^47^ time to nursing home admission,^14^ and survival.^48^ Thus, we conclude that over time courses comparable to that of this study, the trajectory of core features between the 2 disorders can be expected to be similar, which (depending on the nature of the intervention) supports the practice of combining the 2 groups to improve power in studies in which investigators wish to use change in core features as endpoints.

Although average trajectories of symptoms are useful for guiding group-level endpoints, the high interparticipant variability demonstrates the inherent heterogeneity of patients with Lewy body dementia. As a result, such trajectories cannot be applied at the individual level in clinical practice. Furthermore, although the aim here was to use more common and validated tools for exploring such symptoms that are likely to be used as endpoints in clinical trials, the use of composite endpoints (such as NPI-sleep) inherently compromises sensitivity to more specific features of a symptom complex (e.g., daytime somnolence), which may on their own be a marker of progression. Studies exploring more isolated symptoms with richer rating scales designed for such symptoms (e.g., use of Epworth Sleepiness Scale^49^ for daytime somnolence) may be necessary to properly understand the trajectory of individual symptoms. Likewise, other endpoints not used here such as detailed neuropsychological testing (recently found to differentiate PDD and DLB^16^) may also be useful adjuncts for measuring clinical progression across specific symptom domains. Several endpoints were informed by a close informant (mainly partners and close family members), but whether specific features of the informants/caregivers (such as demographics) affect reporting should be considered in future studies. Cognitive endpoints using tests such as the MMSE have been used commonly in many recent disease-modifying trials in AD and have been the focus of most longitudinal studies of the natural history of Lewy body dementia to date.^9,11,14^ However, cognitive measures are dependent on a patient's state on the day of testing and are especially affected by fluctuations in patients with Lewy body dementia.^50^ This was confirmed in our study by the statistically significant degree of individual variability. This argues in favor of the use of semiquantitative instruments (such as the DCFS) that ask the patient and informant to comment on the last month and are therefore less likely to be confounded by such state-dependent variations.

That progression of certain features could not be detected may be a function of the limited duration of the study. However, the relatively rapid progression of this disease is an important consideration, and such a time frame was felt important to accommodate enrollment of individuals with DLB with a diverse range of severity while also ensuring a high rate of completion as seen in our study. Furthermore, the use of a linear mixed modeling approach, which assesses the trajectory and variability of a disease measure, offers potentially greater power for detecting significant differences in symptom progression over time compared to the more traditionally used method in clinical trials of assessing only the absolute difference between baseline and a single point in time at the end of the study. This latter point is especially pertinent to patients with Lewy body dementia who display significant degrees of interindividual and intraindividual variability. Such considerations will also be relevant in future studies, especially clinical trials, in which duration and sample size are often also dictated by funding constraints. Indeed, power and sample size calculations are the necessary next step to operationalize our findings. However, outstanding questions remain regarding the minimal clinically important differences for fluctuation and sleep-related measures, as well as the appropriate study design (e.g., comparing absolute change at 6 months and change in trajectories using linear mixed modeling approaches). Future studies involving validation of our results in larger samples, use of caregiver-reported scales to support minimal clinically important differences, and power calculations derived with simulation-based approaches will be essential to facilitate planning of clinical trials in DLB.

Last, as with most clinical trials, demographic data were not collected for those who declined participation (38%). Years of education was also not captured in this study. Care should be taken when generalizing to patients in other countries with potentially different practice patterns and educational, racial, and ethnic backgrounds.

Lewy body dementias are heterogeneous disorders, and defining disease progression through clinical measures remains an urgent challenge for the field. The detection and quantification of a significant change in the trajectory of key clinical features and symptoms over a relatively short time frame, beyond just cognitive decline, suggest the feasibility of using some of these features as clinical endpoints in isolation or as part of a composite measure in therapeutic trials. These findings also reinforce the Lewy body dementias as a tractable disease model for testing disease-modifying interventions.

## Glossary

AD: Alzheimer disease
CI: confidence interval
DCFS: Dementia Cognitive Fluctuations Scale
DIAMOND: Diagnosis and Management of Neurodegenerative Dementia
DLB: dementia with Lewy bodies
MMSE: Mini-Mental State Examination
MoCA: Montreal Cognitive Assessment
NHS: National Health Service
NPI: Neuropsychiatric Inventory
PDD: Parkinson disease dementia
UPDRS: Unified Parkinson's Disease Rating Scale
